# The Effect of Carob (Ceratonia Siliqua) on Sperm Parameters of Infertile Men: Systematic Review and Meta-Analysis of Randomized Controlled Trials

**DOI:** 10.1007/s43032-024-01534-7

**Published:** 2024-04-18

**Authors:** Aysu Yıldız Karaahmet, Nuran Gençtürk, Emine Kınık

**Affiliations:** 1 Faculty of Health Sciences, Midwifery Department, Halic University, Istanbul, Türkiye; 2 Faculty of Health Sciences, Department of Midwifery, Istanbul University-Cerrahpaşa, Istanbul, Türkiye; 3 Graduate Education Institute, Department of Midwifery, Istanbul University-Cerrahpaşa, Istanbul, Turkey

**Keywords:** Ceratonia siliqua, Male infertility, Meta-analysis, Sperm characteristics, Sperm parameters

## Abstract

Carob (Ceratonia siliqua) supplements can increase sperm quality. This study aimed to summarize the available evidence about the effects of carob (Ceratonia siliqua) supplements on sperm quality and reproductive hormones in infertile men. Systematic searches of five databases were conducted from inception to October 20, with the last update on November 20, 2023. Randomized clinical trials (RCTs) that compared carob (Ceratonia siliqua) supplements with nonintervention control groups on infertile man. Risk of bias and certainty of evidence were assessed by the Cochrane risk of bias tool 2. Summary effect size measures were calculated using a random-effects model estimation and are reported as standardized mean differences and 95% confidence intervals. Reporting followed the PRISMA guidelines. The analysis included four studies involving 236 infertile men. It was found that sperm motility of infertile men improved after carob intervention (MD:11.30, 95% CI:5.97 to 16.64, Z = 4.15, *p* < 0.00001), and there was a significant difference compared to control groups. The effect size of carob on semen quantity in infertile men was positive, and the relationship was statistically significant (MD:5.42, 95% CI:1.58 to 9.42, Z = 2.77, *p* = 0.006). When hormone parameters of infertile men were analyzed, the MDA (malondialdehyde) value decreased compared to the control group (MD = -4.81, 95% CI: -10.18 to 0.56, Z = 1.76, *p* = 0.08), and there was a significant difference between them. Carob (Ceratonia siliqua) supplements was associated with improvement in sperm quality compared with nonintervention control groups in infertile man. However, high-quality, larger RCTs are required to draw more definitive conclusions.

## Introduction

Infertility has been defined as a global health problem by the World Health Organisation (WHO). IWhile it is estimated that 186 million people worldwide have infertility problems, this rate shows that one in every person in the world has an infertility problem [1].

Clinical infertility is defined as the inability of a couple to become pregnant despite 12 months of trying. It is estimated that approximately 10–20% of couples in the world are infertile. The concept of reproductive success is evaluated and defined at the couple level. As a result, the etiology of infertility in a couple is reported to be caused by one or both members of the couple. Most studies estimate that the male factor is associated with 25% of infertility cases [2, 3]. Male fertility is most commonly defined by semen quality. Clinical guidelines state that semen analysis should be included in the initial examination of an infertile couple. The presence of the male factor is often based on abnormal sperm parameters [4]. Impairment in sperm production and function and damage to the spermatogenesis process are among the most common causes of male infertility [5].

Studies have shown that infertile men have a lower antioxidant capacity than fertile men. Medicinal plants with antioxidant capacity can cope with the destructive effects of free radicals produced and oxidative stress in them can minimize cell in infertile men [5, 6]. Carob tree (Ceratonia siliqua L.), belonging to the Fabaceae family, grows in Italy, Iran and Turkey. The plant, traditionally used to improve semen quality in some countries such as Iran and Turkey, is a natural source of antioxidants [7]. Ceratonia siliqua (C. siliqua) has strong antioxidant features and contains many minerals and vitamins as well as polyphenol. Phenolic antioxidants have improvement effect on oxidative stress pathology, that have little side effect, affordable and easy to reach. In addition, C. siliqua contains vitamins C, D, E, niacin and folic as well as many minerals such as calcium, potassium, sodium phosphorus, and iron. The effect of carob on male infertility has been demonstrated in animal and human studies [8]. A small number of studies have shown that carob extract improves semen quality and increases testosterone levels [9, 10]. The focus of this study is to conduct a systematic review and meta-analysis of the available evidence on the effect of carob (Ceratonia siliqua) supplements on sperm quality and parameters in infertile men.

### Research Questions

1. What is the effect of carob supplements on sperm motility in the treatment of male infertility?
2. How do carob supplements affect sperm count in male infertility treatment?
3. What is the effect of carob supplements on sperm volume in the treatment of male infertility?
4. What is the effect of carob supplements on reproductive hormones in the treatment of male infertility?

## Methods

A systematic review and meta-analysis of studies evaluating the effect of carob supplements given to infertile men on sperm quality and sperm parameters were performed. The PRISMA (Preferred Reporting Items for Systematic Reviews and Meta-Analysis Statement) guidelines were followed in preparing the systematic review and meta-analysis. This systematic review and meta-analysis protocol was registered in the PROSPERO database (PROSPERO Registration Number: CRD42023420489). Two investigators conducted an independent literature review, article selection, data extraction, and quality assessment of all the included articles to ensure unbiased results. If there were any disagreements, all the investigators met to discuss and reach a final consensus. No deviations from the protocol were required during the study, and it was completed by the protocol entered in the PROSPERO database.

### Research Strategy

A literature search for this systematic review was performed between October 20 and November 20, 2023, using five electronic databases: Pubmed (MEDLINE), Cochrane, Google Scholar, Scopus and Web of Science. An article search was performed using MeSH-based keywords. Search terms used were: “infertility” OR “male infertility” AND “male” OR “man” AND “Ceratonia siliqua” OR “carob” OR “sperm parameters” OR “sperm characteristics AND (“Randomised Controlled Trial” OR “Controlled Clinical Trial.” The search strategy was changed according to the characteristics of each database.

### Inclusion Criteria

The following criteria (PICOS) were considered in selecting the studies included in the research.

Participants (P): Infertile males. The following criteria were required for the inclusion of infertile men in the study: (1) aged 20–45 years, with a history of infertility complaints for at least 12 months, and no record of surgical or medical treatments for infertility; (2) having a BMI less than 30; (3) exhibiting oligo-spermatozoa according to WHO criteria (sperm concentration less than 15 million per ml, type A motility less than 25%, and type B mobility less than 50%, normal morphology less than 15%).

Intervention (I): Carob (Ceratonia siliqua) supplements.

Comparison (C): Infertile man without carob interventions.

Outcomes (O): Primary outcomes: Sperm characteristics. Secondary outcomes: (1) Reproductive hormone parameters.

Study design (S): Prospective randomized blind clinical study, double-blind randomized controlled study and randomized controlled trials were included.

Quasi-experimental studies, reviews, case reports, qualitative studies, theses, congress papers, and descriptive studies constituted the study’s exclusion criteria.

### Selection of Studies and Data Extraction

Data for each study were extracted into a predefined Microsoft Excel (V2201, Microsoft, Washington, USA) template under the categories of study details, participants’ characteristics, study protocol, objective measurement tools, subjective measurement tools, and sperm characteristics outcome. A reviewer (E.K.) extracted data using a data extraction form and checked by a second reviewer (A.Y.K.). Disagreements between the two were resolved with a third researcher (N.G.). General characteristics of the study (author, country, year of publication, and study design); characteristics of the study participants (mean age, duration of infertility, and sample size); type and description of the intervention; involvement of health professionals; setting, format, duration, and number of sessions; frequency of contact; duration of follow-up; dropout rate; and main outcome variables for each included study are included in Table 1.

**Table 1:** Characteristics of the participants of the included studies (*n* = 4)

References, Country		Study Design		Study Period		Data Collection Tools		Age		Sample size		Semen profile
Aghajani et al. 2021, Iran [8]		RCT		April 2018 to March 2019		Carob syrup twice a day or vitamin E 100 mg twice a day for 3 months Sperm parameters (count, motility, and morphology), hormonal levels, stress oxidative markers measured after the 3 months in each group		20–45		60 infertile male Intervention Carob syrup group: 30 Control Vit-E group: 30		**Intervention group Carob** **Pre-treatment:** Volume: 2.76 ± 1.62 Count (10^6^/ml): 22.34 ± 19.31 Morphology (%): 8.93 ± 9.52 Total motility (%) 36.76 ± 20.89 **Post-treatment:**Volume: 2.82 ± 1.41 Count (10^6^/ml): 41.87 ± 30.59 Morphology (%):11.36 ± 9.22 Total motility (%):46.72 ± 17.84 **Control group Vit-E** **Pre-treatment:** Volume: 2.20 ± 0.96 Count (10^6^/ml): 23.36 ± 19.96 Morphology (%):8.69 ± 7.28 Total motility (%):32.49 ± 19.32 **Post-treatment:**Volume: 2.48 ± 1.01 Count (10^6^/ml): 27.28 ± 25.98 Morphology (%):4.50 ± 4.71 Total motility (%):29.48 ± 20.22
Sanagoo et al. 2021, Iran [9]		RCT		March 2020		Carob capsules 500 mg three times a day for 90 days or vitamin E capsules three times a day for 90 days Sperm parameters (count, motility, and morphology) were measured after the 3 months in each group		20–45		50 infertil male Intervention Carob capsules group: 25 Control Vit-E group: 25		**Intervention group Carob** **Pre-treatment:** Count: 49.08 to million/mL Total motility (%):45.90 Morphology (%):11.52 **Post-treatment:** Count: 60.22 million/mL Total motility (%):67.05 Morphology (%):52.90 **Control group Vit-E** **Pre-treatment:** Count: 47.64 to million/mL Total motility (%):55.23 Morphology (%):10.20 **Post-treatment:** Count: 58.88 million/mL Total motility (%):38.10 Morphology (%):77.47
Pilehvari et al. 2023, Iran [10]	RCT		May 2019 to September 2019		Carob 1.5 g daily for 12 weeks The received placebo contained lactose for 12 weeks Sperm parameters (count, motility, and morphology) were measured after the 12 weeks in each group Testosterone, prolactin, LH, FSH and TSH were performed before and after drug treatment in two groups. Sexual function was assessed in the groups in two stages before and after the intervention using the standard International Index of Erectile Function		Under the age of 40		60 infertile male Intervention carob group: 30 Control placebo group: 30		**Intervention group Carob** **Pre-treatment:** Count: 25 million/mL Total motility (%): 14 Morphology (%): 26 Volume: 26 **Post-treatment:** Count: 23 million/mL Total motility (%): 20 Morphology (%): 27 Volume: 25 **Control group Placebo** **Pre-treatment:** Count: 16 million/mL Total motility (%): 14 Morphology (%): 21 Volume: 18 **Post-treatment:** Count: 18 million/mL Total motility (%): 14 Morphology (%): 19 Volume: 21	
Ahmadian et al. 2023, Iran [11]	RCT		March 2019		Carob capsules 400 mg twice a daily for 12 weeks Pentoxifylline capsules 400 mg twice a daily for 12 weeks 30-min acupuncture sessions for 12 weeks. (the first month, two sessions per week; the next 2 months, one session per week, and 16 sessions) All subjects were examined before entering the study and at the end of the intervention regarding sperm components, serum levels of sex hormones, and the number of inflammatory factors		20–45		66 infertile male Intervention carob group: 22 acupuncture group: 22 Control pentoxifylline group: 22		Comparison of sperm motility between and within study groups **Mean changes (Mean**_**after**_** − Mean**_**before**_**) sperm motility** Carob group: 11.91 Pentoxifylline group: 3.92 Acupuncture group: 10.28	

**RCT:** Randomize Control Trial**, FSH:** Follicle-stimulating hormone **LH**: Luteinizan Hormone, **TSH:** Tiroit Stimulating Hormone

### Risk of Bias Assessment

The Cochrane Risk-of-Bias tool for randomized controlled trials (RoB-2) version 2 was used to assess the methodological quality of each included study. The risk of bias was evaluated for seven areas: randomization, allocation concealment, blinding of participants and staff, blinding of outcome assessment, missing outcome data, selective reporting, and other biases. In addition to these areas, this study also considered other sources of bias, such as fundamental imbalances between groups and potential confounding factors. Each study’s risk of bias error was categorized as low, high, or uncertain. Two investigators (A.Y.K. and E.K.) conducted the risk of bias assessment independently, and in case of disagreement, they reviewed the full text together to reach a consensus.

### Statistical Analysis

We conducted meta-analyses using continuous data and random-effects models to compare carob supplements with control groups on sperm quality outcomes using the data from the included studies. Because the participant mixes in the included studies differed, the random-effects model analysis was used. The carob effect was measured using mean difference (MD) with 95% confidence intervals (95% CI), as the included studies presented sperm quality using same methods. MD was interpreted per Cohen thresholds: trivial (< 0.2), small (0.2 to < 0.5), medium (0.5 to < 0.8), and large (> 0.8).As reported in the original studies, the mean difference and standard deviation of sperm quality outcomes for carob and control groups were entered into the Review Manager software (RevMan, version 5.4; The Cochrane Collaboration, Copenhagen, Denmark). However, because most of the included studies did not report the mean difference and standard deviation for sperm quality outcomes in carob and control groups, we requested the information from the corresponding authors. In case of a negative response from the corresponding authors, we calculated the mean difference as postintervention mean value minus baseline mean value, and the standard deviation of the mean difference was determined using the following equation:

$$SD\;mean\;difference\;=\surd\left(SDpre-test\;2\;+\;SDpost-test\;2\right)-\left(2r\;\times SDpre-test\;\times\;SDpost\;-\;test\right)$$

For this equation, we used three r values (0.5, 0.7, and 0.9) for sperm quality outcomes in carob and control groups. These calculations are recommended for performing a metaanalysis of change scores when continuous data are missing. To improve our results, we conducted a preplanned sensitivity analysis (the one study removed method) to assess the impact of each study on the overall results. Publication bias was visually assessed using a preplanned funnel plot analysis by plotting the MD of each trial against its standard error.

Statistical heterogeneity was assessed using Tau squared (*I*^*2*^), Cochran’s Q test, and the inconsistency *I*^*2*^ test. The *I*^*2*^ statistic estimates the degree of heterogeneity in effects across a set of studies between 0 and 100%, with values above 30%, 50%, and 75% indicating moderate, substantial, and high heterogeneity, respectively. All statistical analyses were performed in the Review Manager software (RevMan, version 5.4.1.; The Cochrane Collaboration, Copenhagen, Denmark). The significance level was set at *p* < 0.10 for Q statistic51 and *p* < 0.05 for all other analyses.

## Results

### Included Studies

**Fig. 1:**
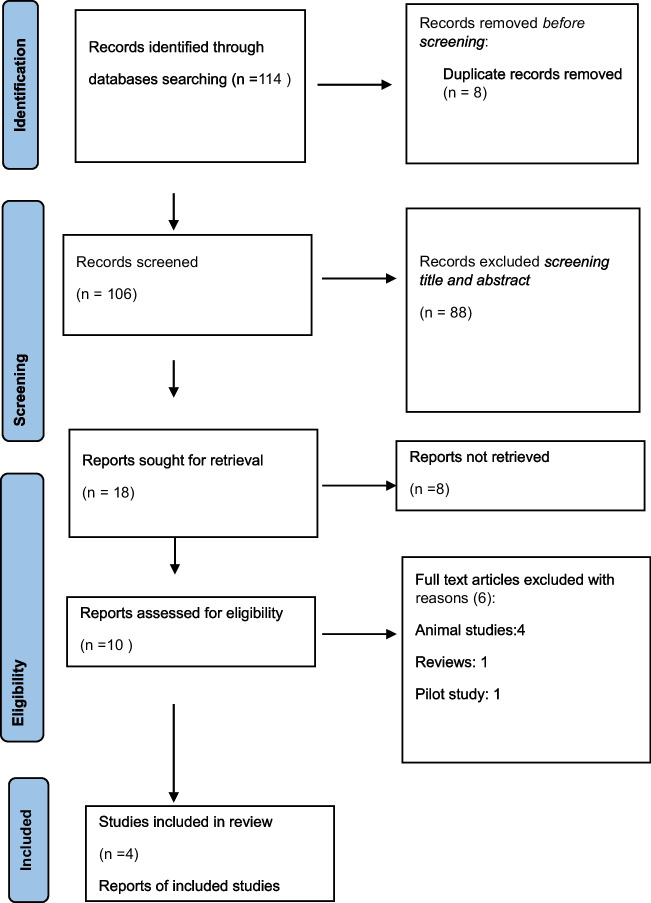
PRISMA flow diagram. PRISMA: Preferred Reporting Items for Systematic Reviews and Meta-Analyses

Figure 1 presents the PRISMA flowchart summarising the literature search and study selection process. The electronic database search and manual search yielded a total of 114 articles. After eliminating duplicate records, 106 articles were included. Titles and abstracts were read to identify relevant articles, review articles, protocols, duplications, and different populations, and 88 articles were excluded because they did not meet the inclusion criteria. The remaining 18 full-text articles were assessed for eligibility, and four articles that met the criteria for Randomised Controlled Trials (RCTs) were included in the analysis (see Fig. 1).

### Study Features

This meta-analysis and systematic review involved four studies and a total of 236 infertile men. The objective was to analyze the impact of carob supplements on the sperm quality and sperm parameters of infertile men. All studies were conducted in Iran, including Aghajani et al. [8], Sanagoo et al. [9], Pilehvari et al. [10] and Ahmadian et al. [11]. None of the studies from other countries met the relevant criteria. The study design for all included studies was Randomized Controlled Trials (RCTs). In all studies, carob supplements were administered to infertile men, and sperm quality and sperm parameters were assessed as the primary outcome. Table 1 provides a summary of the study characteristics.

### Outcomes: Primary Outcomes: Semen Quality

#### Semen Motility

**Fig. 2:**
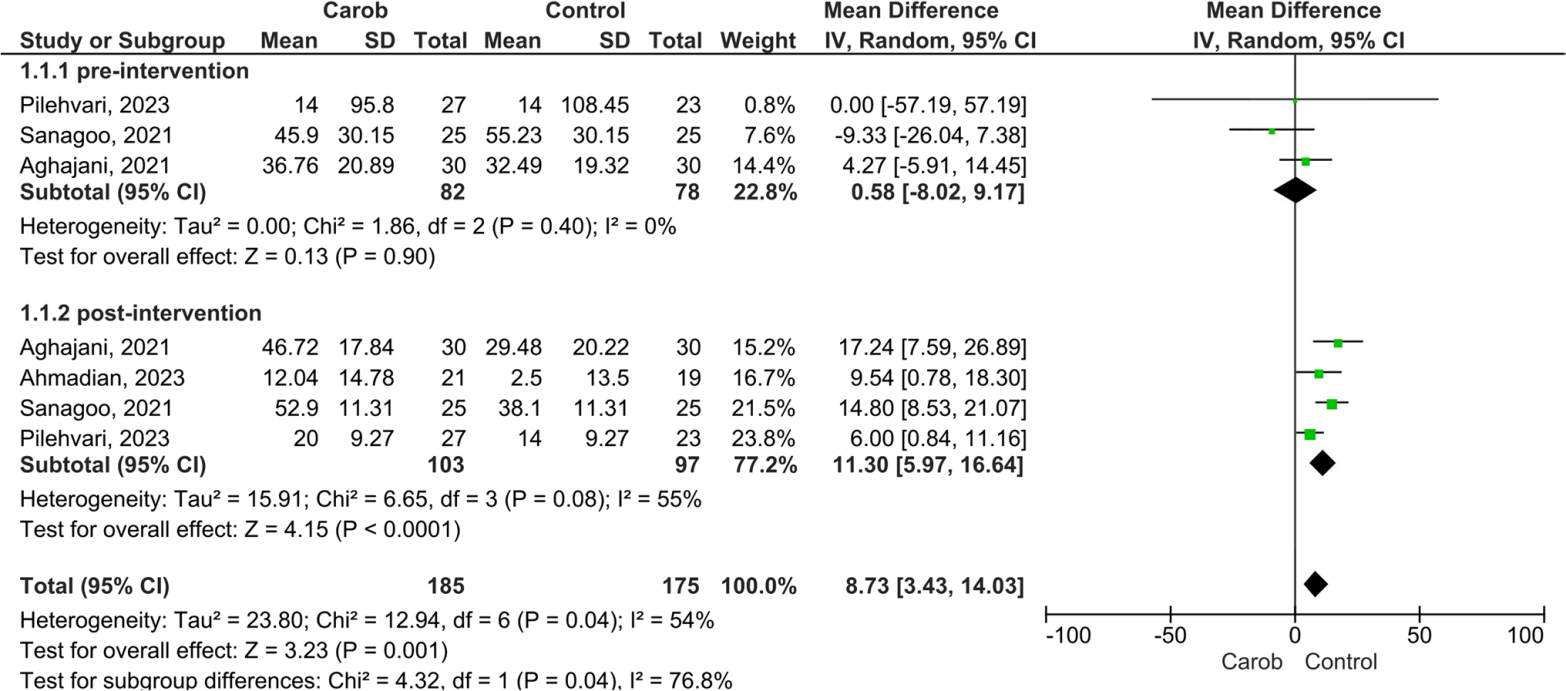
Meta-analysis Results on the Effect of Carob on Semen Motility in Infertile Men **a**) Pre-intervention, **b**) Post-intervention

In Fig. 2, the results of the meta-analysis of the effect of carob intervention on semen motility in infertile men are presented as a forest plot. All included studies reported results on the effect of carob on semen motility. When the pre-intervention semen motility of men in these studies was considered, there was no heterogeneity between them (I^2^ = 0%, *p* = 0.40). The mean random effects model showed no significant difference between the groups on pre-intervention semen motility (MD:0.58, 95% CI:-8.02 to 9.17, Z = 0.13, *p* = 0.90) (Fig. 2). A moderate level of heterogeneity was found when pooling the data after carob intervention (I2 = 55%, *p* = 0.08). Thus, the mean random effects model showed that there was a significant difference between the groups on semen motility after the intervention, and semen motility was positively affected (MD:11.30, 95% CI:5.97 to 16.64, Z = 4.15, *p* < 0.00001) (Fig. 2).

### Semen Volume

**Fig. 3:**
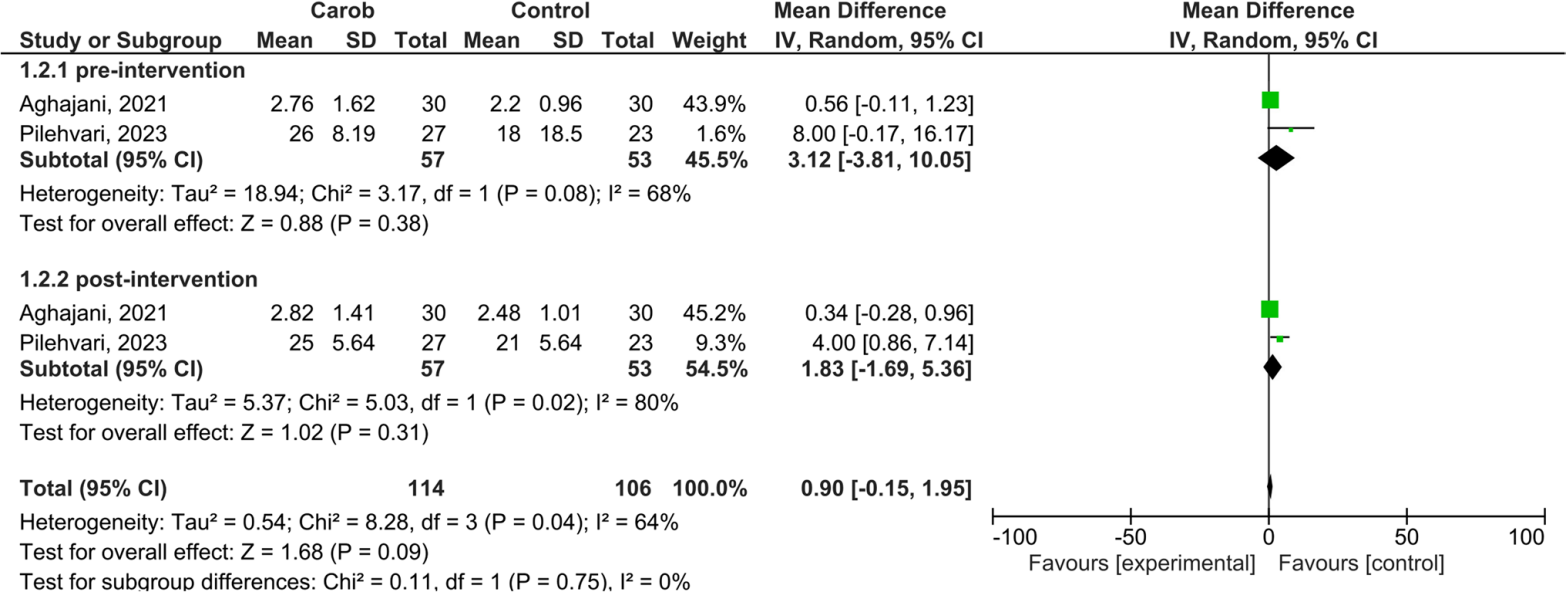
Meta-analysis Results on the Effect of Carob on Semen Volume in Infertile Men **a**) Pre-intervention, **b**) Post-intervention

In Fig. 3, the results of the meta-analysis of the effect of carob intervention on semen volume in infertile men are presented as a forest plot. Only two included studies reported results on the effect of carob on semen volume. In these studies, there was a high level of heterogeneity between them when men’s pre-intervention semen volume was considered (I^2^ = 68%, *p* = 0.08). The mean random effects model showed no significant difference between the groups on pre-intervention semen volume (MD:3.12, 95% CI:-3.81 to 10.05, Z = 0.88, *p* = 0.38) (Fig. 3). Heterogeneity was again high when data pooling after carob intervention (I2 = 80%, *p* = 0.002). The pooled effect of carob on semen volume was positive with a small effect size, but the association was not statistically significant (MD:1.83, 95% CI:-1.69 to 5.36, Z = 1.02, *p* = 0.31) (Fig. 3). Despite the wide range of effect sizes, positive associations between carob and semen volume were observed in all individual studies.

### Semen Count

**Fig. 4:**
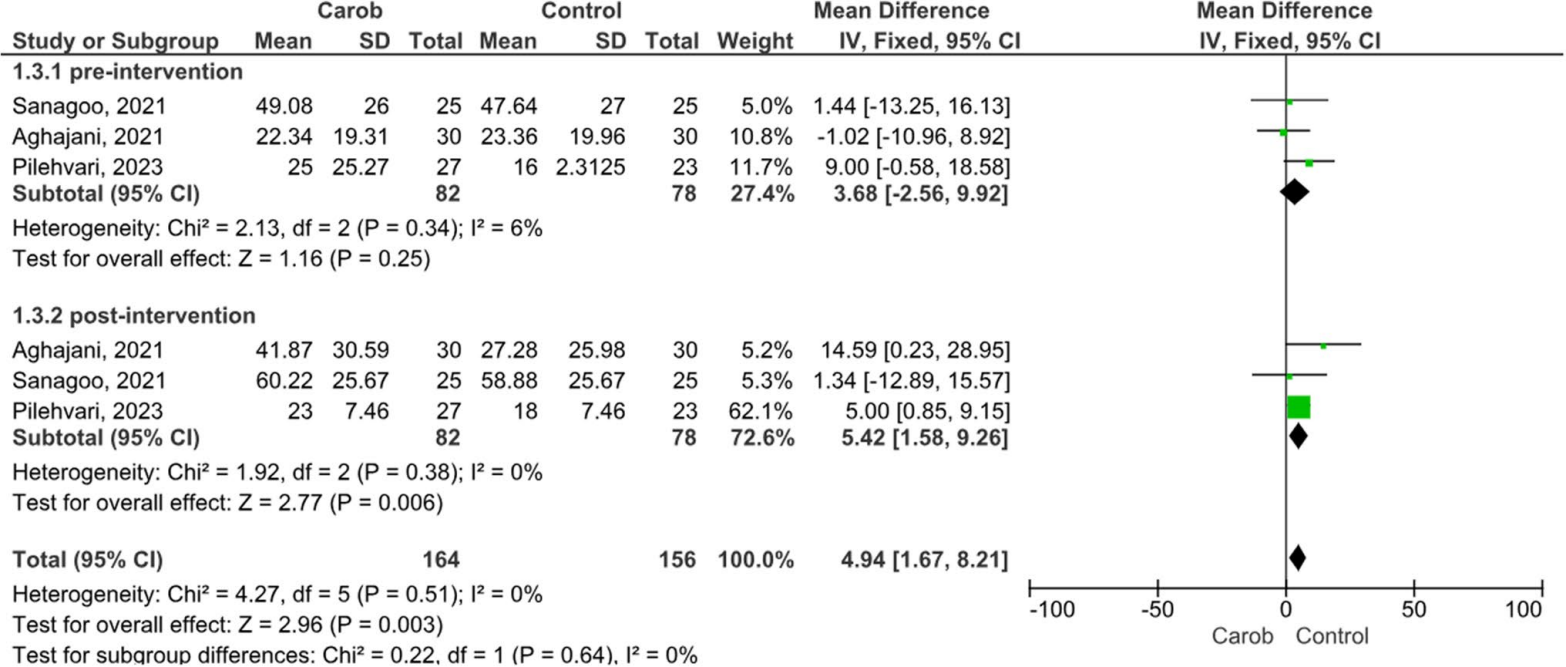
Meta-analysis Results on the Effect of Carob on Semen Count in Infertile Men **a**) Pre-intervention, **b**) Post-intervention

In Fig. 4, the results of the meta-analysis of the effect of carob intervention on semen count in infertile men are presented as a forest plot. Three included studies reported results on the effect of carob on semen count. These studies showed moderate heterogeneity between them when men’s pre-intervention semen count was considered (I^2^ = 46%, *p* = 0.16). The mean random effects model showed no significant difference between the groups on pre-intervention semen count (MD:3.68, 95% CI: 2.56 to 9.92, Z = 1.16, *p* = 0.25) (Fig. 4). There was no heterogeneity in semen quantity data after carob intervention (I^2^ = 0%, *p* = 0.38). The association with the effect size of carob on semen quantity was positive and statistically significant (MD:5.42, 95% CI:1.58 to 9.42, Z = 2.77, *p* = 0.006) (Fig. 4).

### Secondary Outcome

#### Hormonal Parameters

**Fig. 5:**
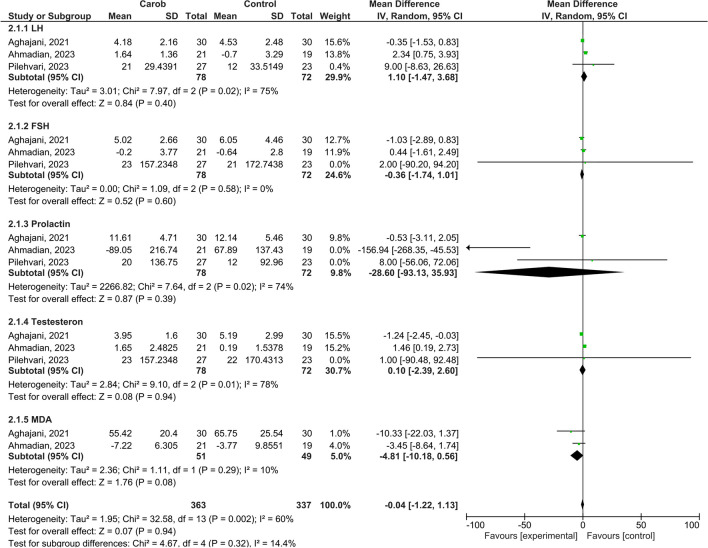
Meta-analysis Results on the Effect of Carob on Hormone Parameters in Infertile Men 2.1.1) LH, 2.1.2) FSH, 2.1.3) Prolactin, 2.1.4) Testosterone, 2.1.5.) MDA

Meta-analyses did not reveal any significant differences between men in the carob and control groups on hormone parameters except for MDA (MD = -4.81, 95% CI: -10.18 to 0.56, Z = 1.76, *p* = 0.08) (*p* > 0.05) (Fig. 5). Prolactin areas showed the greatest effect (MD = -28.60, 95% CI: -93.13 to 35). According to Cohen’s classification, the pooled effect sizes of LH (Luteinizing Hormone), FSH (Follicle Stimulating Hormone), Testosterone and total hormone parameter domains were all small (MD = 1.10, 95% CI: -1.47 to 3.68, MD = -0.37, 95% CI: -1.74 to 1.01, MD = 0.10; 95% CI: -2.39 to 2.60 respectively) (Fig. 3).

**Fig. 6:**
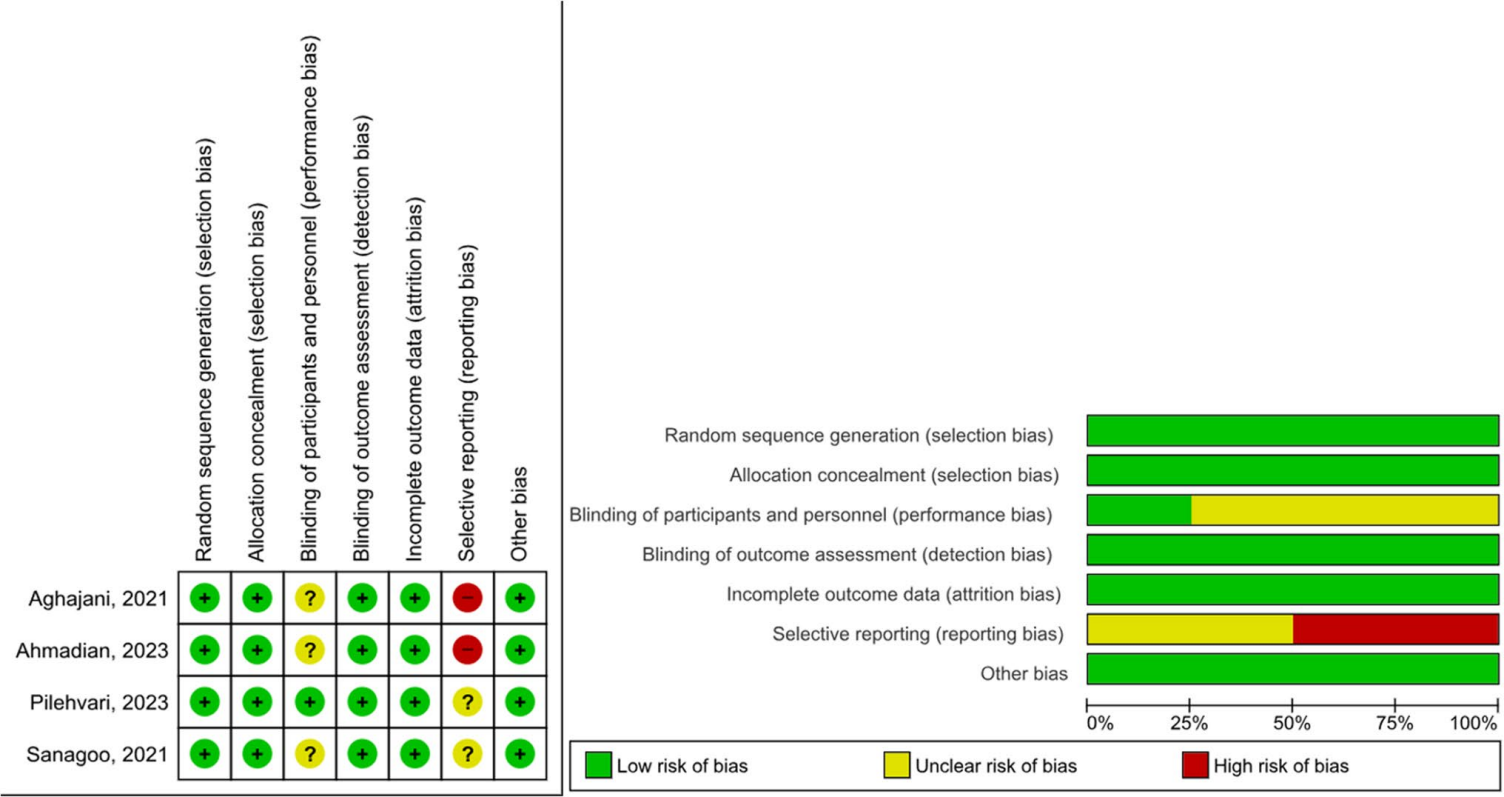
Risk of bias graph

#### Risk of Bias

All of the studies included in the meta-analysis had a proper method for randomly assigning participants to the cognitive behavioral intervention groups. As a result, the studies were considered at low risk of favoritism error. Additionally, all of the studies ensured confidentiality of the allocation for randomization and were therefore assessed as being at low risk of favoritism error. However, three studies [8, 9, 11] were evaluated to be at risk of unclear bias because they did not provide information about the blinding of trial participants and investigators to the study. It was found that none of the studies included in the meta-analysis used blind outcome assessment and were judged to have a low risk of bias error. In all studies, drop-outs were evenly distributed between the intervention and control groups or were deemed to have a low risk of attrition due to a minimal amount of drop-outs that would not affect the study results (Fig. 6).

Two studies included in the meta-analysis [8, 11] were assessed as high bias in their methods, as they discussed important reported outcomes, including adverse outcomes, and matched those reported in their registries, while two studies [9, 10] were assessed at risk of unclear bias. For each included study, significant concerns were described regarding other possible sources of bias not previously addressed in the above categories. In particular, a conflict of interest declaration and a funding source were sought. Not all of the included studies reported any other risk of bias.

## Discussion

This systematic review and meta-analysis aimed to review the available studies on the effect of carob supplements (Ceratonia siliqua) on semen quality and parameters in infertile men. Four studies determining the effect of carob on semen quality in infertile men were included in the review. As seen in the studies reviewed in this systematic review, studies have found that carob positively affects semen count, volume, and motility after the intervention. The results of this study indicated that carob consumption generally improved sperm parameters.

Carob (Ceratonia siliqua L.) is a tree species native to the Mediterranean region belonging to the subfamily Caesalpinaceae of the family Leguminoseae (Fabaceae-Legumes). Carob contains polyphenolic compounds, many vitamins, and various minerals. Carob (Ceratonia siliqua) is a natural source of antioxidants and is traditionally used to improve semen quality [12, 13]. In the studies included in this review, the intervention for infertile men was carob supplements. In this meta-analysis, the use of carob supplements positively affected semen motility in infertile men. In all individual studies, there was a positive relationship and statistical significance between semen volume, semen quantity, and the use of carob supplements. Limited evidence has shown that carob extract improves semen quality and raises testosterone levels; it also improves biochemical parameters that affect Leydig cells and testosterone biosynthesis, thus affecting sex hormones, sperm production, and the spermatogenesis process [14, 15].

So far, human and animal studies on the effects of carob on sperm quality and parameters have been conducted. In the animal study conducted by Ghorbaninejad et al. in 2023, it was reported that carob extract affected the spermatogenesis process in infertile mice and increased sperm production[16]. The results of the study conducted by Nemati et al. on roosters and Ghorbaninejad et al. are similar [17]. In the study conducted by ElAkhdar et al. on infertile mice, it was reported that carob increased FSH, LH, and testosterone levels and positively affected the spermatogenesis process [18].

Some in vitro studies have also shown the positive effect of carob extract on sperm parameters. These studies confirm the positive effect of carob capsules on increasing the amount of sex hormones and sperm motility [18, 19]. However, there have not been sufficient animal and human studies on the effects of the extract of this plant and the amount of effective use [9]. However, the fact that the control groups of the studies included in the meta-analysis are different from each other may cause heterogeneity and this reduced the consistency of the carob intervention, but we reduced this misconception with the random effect model we used.

After reviewing the literature, we found no meta-analytical studies that examined the effect of carob supplements on semen parameters in infertile men for comparison with the findings of this meta-analysis. Only randomized controlled trials were chosen to ensure consistency in the methods used across the studies included in the analysis. These studies are known for minimizing bias and are considered the most reliable form of evidence-based practice. By selecting only men who exhibited symptoms of infertility, we were able to determine the effect of carob on male infertility accurately.

### Limitations of the Research

This study has some strengths and limitations. One strength is that it analyzed up-to-date studies and had access to extensive scanning resources. However, the limitation of the study is that it only considered articles published in English from a single country (Iran), which means that studies published in other languages were not included. The limitations are that some randomized controlled trials were conducted with a small sample size, and carob supplements were administered in different doses and forms. Studies have also included different interventions in control groups. This raises doubts about reducing the consistency of the carob intervention.This systematic review and meta-analysis study focused on human studies. Therefore, a systematic review and meta-analysis of animal studies in this field is recommended.

## Conclusions

The results of this systematic review and meta-analysis showed that the use of carob supplements in infertile men improved sperm quality and parameters. However, since the sample size of some studies was small, it seems necessary to conduct randomized controlled clinical trials designed with an appropriate sample size to determine the effect of this herb on sperm parameters. At the same time, it is recommended to conduct placebo-controlled studies in order not to affect the consistency of the carob intervention in the control groups of the studies included in the study.

## Data Availability

Not applicable.

## References

[1] World Health Organization. Infertility prevalence estimates: 1990–2021. 2023. https://www.who.int/publications/i/item/978920068315. Accessed 31.10.2023.

[2] American Urological Association. Male infertility. 2020. https://www.auanet.org/meetings-and-education/for-medical-students/medical-students-curriculum/male-infertility. Accessed 23.10.2023.

[3] Roozbeh N, Amirian A, Abdi F, Haghdoost S. A systematic review on use of medicinal plants for male infertility treatment. J Family Reprod Health. 2021;15(2):74. 34721595 10.18502/jfrh.v15i2.6447 PMC8520662

[4] Eisenberg ML, Esteves SC, Lamb DJ, Hotaling JM, Giwercman A, Hwang K, Cheng YS. Male infertility. Nature Rev Disease Prim. 2023;9(1):49. 37709866 10.1038/s41572-023-00459-w

[5] Abdi F, Roozbeh N, Mortazavian AM. Effects of date palm pollen on fertility: research proposal for a systematic review. BMC Res Notes. 2017;10(1):1–4. 28764804 10.1186/s13104-017-2697-3 PMC5540518

[6] Vafaei A, Mohammadi S, Fazel A, Soukhtanloo M, Mohammadipour A, Beheshti F. Effects of carob (Ceratonia siliqua) on sperm quality, testicular structure, testosterone level and oxidative stress in busulfan-induced infertile mice. Pharmaceut Sci. 2018;24(2):104–11.

[7] Ata A, Yildiz-Gulay O, Güngör S, Balic A, Gulay MS. The effect of carob (Ceratonia siliqua) bean extract on male New Zealand White rabbit semen. World Rabbit Sci. 2018;26(3):209–15. 10.4995/wrs.2018.10154

[8] Aghajani MMR, Mahjoub S, Mojab F, Namdari M, Gorji NM, Dashtaki A, Mirabi P. Comparison of the effect of Ceratonia siliqua L.(carob) syrup and vitamin E on sperm parameters, oxidative stress index, and sex hormones in infertile men: A randomized controlled trial. Reprod Sci. 2021;28(3):766–74. 32959223 10.1007/s43032-020-00314-3

[9] Sanagoo S, Farshbaf-Khalili A, Asgharian P, Hazhir S, Oskouei BS. Comparison of the effect of Ceratonia siliqua L. fruit oral capsule and vitamin E on semen parameters in men with idiopathic infertility: a triple-blind randomized controlled clinical trial. J Complement Integr Med. 2021;18(4):791–6. 34704430 10.1515/jcim-2020-0095

[10] Pilehvari S, Bahar TG, Masoumi SZ, Kazemi F, Moradkhani S, Haghi AR, Maleki P. Effect of carob supplement on spermogram parameters and sexual function of infertile men referred to the Infertility Center, Hamadan, Iran, 2019: a randomized controlled trial. J Fam Reprod Health. 2023;17(3):142–50. 10.18502/jfrh.v17i3.13537 PMC11070741 38716296

[11] Ahmadian M, Salari R, Noras MR, Ahmadnia H, Esmaily H, Bahrami-Taghanaki HR. Comparison of the Effect of Carob Capsule and acupuncture on Sperm Motility, in Idiopathic Male Infertility: A Randomised Controlled Trial. J Herbal Med. 2023;42:100805. 10.1016/j.hermed.2023.100805

[12] Micheletti C, Medori MC, Bonetti G, Iaconelli A, Aquilanti B, Matera G, Bertelli M. Effects of carob extract on the intestinal microbiome and glucose metabolism: a systematic review and meta-analysis. La Clinica Terapeutica, 2023;174(Suppl 2(6)):169–172. 10.7417/CT.2023.2484 37994761

[13] Aghajani MMR, Gorji NM, Mirabi P, Mojab F. Effect of Ceratonia siliqua (Carob) syrup and vitamin E on sperm parameters, oxidative stress index and sex hormones in infertile men: Protocol for a randomized controlled trial. Caspian J Intern Med. 2019;10(4):452. 31814945 10.22088/cjim.10.4.452 PMC6856924

[14] Soleimanzadeh A, Kian M, Moradi S, Mahmoudi S. Carob (Ceratonia siliqua L.) fruit hydro-alcoholic extract alleviates reproductive toxicity of lead in male mice: Evidence on sperm parameters, sex hormones, oxidative stress biomarkers and expression of Nrf2 and iNOS. Avicenna J Phytomed. 2020;10(1):35. 31921606 PMC6941692

[15] Ghorbaninejad Z, Eghbali A, Ghorbaninejad M, Ayyari M, Zuchowski J, Kowalczyk M, Baharvand H, Shahverdi A, Eftekhari-Yazdi P, Esfandiari F. Carob extract induces spermatogenesis in an infertile mouse model via upregulation of Prm1, Plzf, Bcl-6b, Dazl, Ngn3, Stra8, and Smc1b. J Ethnopharmacol. 2023;301:115760. 10.1016/j.jep.2022.115760 36209951

[16] Nemati Z, Dehgani P, Besharati M, Amirdahri S. Dietary carob fruit (Ceratonia siliqua L.) supplementation improves spermatogenesis, semen quality and embryonic death via antioxidant effect in aging broiler breeder roosters. Animal Reprod Sci. 2022;239:106967. 10.1016/j.anireprosci.2022.106967 35299115

[17] ElAkhdar N, Bawab R, Borjac J. Effect of Ceratonia siliqua and Cucurbita pepo seeds extracts on spermatogenesis in male mice. Beirut Arab Univ J-Health Wellbeing. 2020;3(1):7.

[18] Faramarzi A, Aghaz F, Bakhtiari M, Khazaei M. In vitro application of Ceratonia siliqua improved sperm parameters and chromatin quality after vitrifacation in normozoospermic aged men. Middle East Fertil Soc J. 2020;24:1–5. 10.1186/s43043-019-0007-9

[19] Javanmard F, Bidmeshkipour A, Ghasemian Yadegari J. Ceratonia siliqua L improved cryodamage of asthenozoospermic specimens: an experimental study. Intl J Reprod Biomed. 2023;20(12):1029–1038. Published 2023 Jan 9. 10.18502/ijrm.v20i12.12564 10.18502/ijrm.v20i12.12564 PMC9928979 36819209

